# Supplementary material for: The Effects of Differentiated Organic Fertilization on Tomato Production and Phenolic Content in Traditional and High-Yielding Varieties
Source: Antioxidants (Basel). 2022 Oct 28;11(11):2127. doi: 10.3390/antiox11112127 (PMC9687005; doi:10.3390/antiox11112127)
Supplement: Supplementary file 1 [file antioxidants-11-02127-s001.zip › antioxidants-1952141-supplementary.pdf]

Supplementary Data

Table S1. Brix of the tomato samples. Results are the mean of the three replicates ± the standard deviation.

	Varietal group	Variety	ºBrix
1	Pebroter	Cornabel	5.17 ± 0.77 abc
2	Pebroter	Cuban Pepper	5.96 ± 0.61 a
3	Pebroter	Corno Andino	5.60 ± 0.50 ab
4	Montserrat	Montserrat Fitó	5.01 ± 0.71 bcd
5	Montserrat	Can Duran	4.38 ± 0.76 cd
6	Montserrat	Montserrat Ple	4.34 ± 0.55 d

Data are shown as mean ± standard deviation (n=3). Different letters represent significant differences in º Brix observed between varieties ($p < 0.05$).

Table S2. The phenolic compounds identified in the tomato varieties, their neutral molecular formula, the retention time, and the fragments used in the MS.

Compound	Neutral molecular formula	Retention time (min)	MS (Fragments)
Apigenin glucoside	$C_{21}H_{20}O_{10}$	1.08<br>1.16	431/269
Caffeic	$C_9H_8O_4$	1.28	179/135
Caffeic hexoside	$C_{15}H_{18}O_9$	0.83<br>1.00<br>1.08<br>1.30<br>1.36	341/179
Chlorogenic	$C_{16}H_{18}O_9$	1.11	353/191
m-coumaric acid	$C_9H_8O_3$	1.16	163/119
o-coumaric acid	$C_9H_8O_3$	1.65	163/119
p-coumaric acid	$C_9H_8O_3$	0.84	163/119
Coumaric glucoside	$C_{15}H_{18}O_8$	0.85<br>1.16<br>1.28	325/163
Cryptoclorogenic	$C_{16}H_{18}O_9$	1.37	353/191
Ferulic glucoside	$C_{16}H_{20}O_9$	0.98<br>1.10<br>1.30	355/193
Gallic acid	$C_7H_6O_5$	1.68	169/125
Homovanillic acid glucoside	$C_{15}H_{20}O_9$	1.01	343/181
4 -Hydroxybenzoic acid	$C_7H_6O_3$	0.53	137/93
Naringenin	$C_{15}H_{12}O_5$	2.56	271/151
Naringenin glucoside	$C_{21}H_{22}O_{10}$	2.22<br>2.43	433/271
Naringin	$C_{27}H_{32}O_{14}$	2.56	579/271
Neochlorogenic	$C_{16}H_{18}O_9$	1.11	353/191
Protocatechuic	$C_7H_6O_4$	1.13	153/109
Quercetin	$C_{15}H_{10}O_7$	2.58	301/151
Rutin	$C_{27}H_{30}O_{16}$	1.79	609/300
Dicaffeolquinic	$C_{25}H_{24}O_{12}$	2.10<br>2.24	515/353
Tricaffeolquinic	$C_{34}H_{30}O_{15}$	2.49	677/515

Table S3. Polyphenols concentrations (mg/kg) according to fertilization and varieties.

	Fertilization N rich (n=18)			Fertilization N rich+C (n=18)			Fertilization N poor +C (n=18)		
**Polyphenols**	**Total**	**Pebroter**	**Monserrat**	**Total**	**Pebroter**	**Monserrat**	**Total**	**Pebroter**	**Monserrat**
Apigenin glucoside	2.84 ± 1.41	2.35 ± 0.91	3.32 ± 1.69	2.95 ± 1.44	2.18 ± 0.95	3.72 ± 1.48	2.67 ± 1.44	1.96 ± 0.58	3.38 ± 1.71
Caffeic	2.55 ± 1.15	2.22 ± 0.42	2.87 ± 1.55	1.85 ± 0.91	1.87 ± 0.81	1.83 ± 1.06	1.78 ± 0.81	1.78 ± 1.11	1.77 ± 0.39
Caffeic hexoside	9.83 ± 5.14	7.96 ± 3.51	11.69 ± 6.00	9.69 ± 4.22	7.60 ± 4.51	11.78 ± 2.76	6.91 ± 2.85	5.97 ± 1.95	7.85 ± 3.38
Chlorogenic	16.65 ± 5.57	14.41 ± 6.25	18.89 ± 3.93	16.87 ± 8.88	16.12 ± 6.98	17.63 ± 10.85	16.17 ± 8.16	15.42 ± 9.97	16.92 ± 6.38
m-coumaric acid	5.58 ± 2.33	5.24 ± 2.97	5.93 ± 1.57	5.57 ± 1.96	5.59 ± 1.97	5.55 ± 2.07	4.61 ± 1.96	3.89 ± 1.54	5.32 ± 2.16
o-coumaric acid	10.93 ± 6.97	7.55 ± 3.78	14.32 ± 7.96	10.36 ± 5.05	7.25 ± 4.58	13.48 ± 3.37	7.98 ± 4.36	6.16 ± 2.87	9.80 ± 4.98
p-coumaric acid	1.53 ± 1.2	1.14 ± 0.52	1.92 ± 1.56	1.46 ± 0.7	1.39 ± 0.90	1.53 ± 0.46	1.32 ± 0.83	1.02 ± 0.55	1.63 ± 0.98
Coumaric glucoside	22.94 ± 9.07	19.89 ± 8.80	25.99 ± 8.73	22.92 ± 7.37	20.46 ± 7.74	25.39 ± 6.46	18.59 ± 6.63	14.86 ± 5.28	22.31 ± 5.86
Cryptoclorogenic	1.74 ± 0.61	1.68 ± 0.75	1.80 ± 0.47	1.67 ± 0.71	1.68 ± 0.85	1.66 ± 0.59	1.47 ± 0.53	1.41 ± 0.39	1.54 ± 0.66
Ferulic glucoside	1.01 ± 0.52	0.84 ± 0.45	1.17 ± 0.56	1.01 ± 0.58	0.73 ± 0.39	1.30 ± 0.61	0.8 ± 0.38	0.65 ± 0.22	0.95 ± 0.45
Gallic acid	12.52 ± 0.45	12.38 ± 0.43	12.67 ± 0.45	12.68 ± 0.76	12.76 ± 0.91	12.60 ± 0.63	12.46 ± 0.41	12.29 ± 0.40	12.63 ± 0.37
Homovanillic acid glucoside	0.32 ± 0.25	0.25 ± 0.23	0.39 ± 0.26	0.36 ± 0.22	0.23 ± 0.20	0.48 ± 0.18	0.21 ± 0.17	0.10 ± 0.14	0.30 ± 0.14
4 -Hydroxybenzoic acid	0.42 ± 0.26	0.30 ± 0.23	0.54 ± 0.23	0.48 ± 0.24	0.35 ± 0.22	0.61 ± 0.18	0.62 ± 0.26	0.51 ± 0.22	0.72 ± 0.26
Naringenin	14.2 ± 19.49	16.24 ± 19.95	12.17 ± 19.99	8.61 ± 14.71	17.14 ± 17.20	0.08 ± 0.24	23.45 ± 30.2	33.07 ± 34.90	13.82 ± 22.64
Naringenin glucoside	5.03 ± 6.41	6.72 ± 7.65	3.34 ± 4.72	5.5 ± 9.81	10.91 ± 11.77	0.10 ± 0.30	6.46 ± 5.94	10.73 ± 4.75	2.20 ± 3.40
Naringin	0.15 ± 0.45	0.30 ± 0.62	0.00 ± 0.00	0 ± 0	0.00 ± 0.00	0.00 ± 0.00	0.5 ± 0.99	0.52 ± 1.01	0.49 ± 1.03
Neochlorogenic	0.13 ± 0.08	0.11 ± 0.04	0.15 ± 0.10	0.11 ± 0.06	0.10 ± 0.05	0.12 ± 0.06	0.09 ± 0.04	0.08 ± 0.04	0.10 ± 0.03
Protocatechuic	0.87 ± 0.34	0.96 ± 0.41	0.79 ± 0.24	0.81 ± 0.39	0.86 ± 0.37	0.77 ± 0.42	0.93 ± 0.56	1.02 ± 0.75	0.84 ± 0.31
Quercetin	0.67 ± 0.87	0.97 ± 0.95	0.36 ± 0.72	0.39 ± 1	0.77 ± 1.33	0.00 ± 0.00	0.8 ± 0.93	1.23 ± 0.94	0.37 ± 0.74
Rutin	14.01 ± 10.91	14.03 ± 10.52	13.99 ± 11.92	12.37 ± 9.83	16.43 ± 9.23	8.31 ± 9.11	16.08 ± 10.3	20.17 ± 7.66	12.00 ± 11.36
Tricaffeolquinic	0.03 ± 0.06	0.05 ± 0.07	0.02 ± 0.04	0.04 ± 0.06	0.04 ± 0.08	0.05 ± 0.06	0.03 ± 0.06	0.01 ± 0.04	0.05 ± 0.06

Data are shown as mean ± standard deviation.
